# Application of Overall Dynamic Body Acceleration as a Proxy for Estimating the Energy Expenditure of Grazing Farm Animals: Relationship with Heart Rate

**DOI:** 10.1371/journal.pone.0128042

**Published:** 2015-06-01

**Authors:** Masafumi Miwa, Kazato Oishi, Yasuhiro Nakagawa, Hiromichi Maeno, Hiroki Anzai, Hajime Kumagai, Kanji Okano, Hisaya Tobioka, Hiroyuki Hirooka

**Affiliations:** 1 Laboratory of Animal Husbandry Resources, Division of Applied Biosciences, Graduate School of Agriculture, Kyoto University, 606 8502 Kyoto, Japan; 2 Animal Science Laboratory, Department of Biological Resources Management, School of Environmental Science, The University of Shiga Prefecture, 522 8533 Shiga, Japan; 3 Laboratory of Animal Nutrition and Feeding, Department of Animal Science, School of Agriculture, Tokai University, 869 1404 Kumamoto, Japan; Institut Pluridisciplinaire Hubert Curien, FRANCE

## Abstract

Estimating the energy expenditure of farm animals at pasture is important for efficient animal management. In recent years, an alternative technique for estimating energy expenditure by measuring body acceleration has been widely performed in wildlife and human studies, but the availability of the technique in farm animals has not yet been examined. In the present study, we tested the potential use of an acceleration index, overall dynamic body acceleration (ODBA), as a new proxy for estimating the energy expenditure of grazing farm animals (cattle, goats and sheep) at pasture with the simultaneous evaluation of a conventional proxy, heart rate. Body accelerations in three axes and heart rate for cows (n = 8, two breeds), goats (n = 6) and sheep (n = 5) were recorded, and the effect of ODBA calculated from the body accelerations on heart rate was analyzed. In addition, the effects of the two other activity indices, the number of steps and vectorial dynamic body acceleration (VeDBA), on heart rate were also investigated. The results of the comparison among three activity indices indicated that ODBA was the best predictor for heart rate. Although the relationship between ODBA and heart rate was different between the groups of species and breeds and between individuals (*P*<0.01), the difference could be explained by different body weights; a common equation could be established by correcting the body weights (*M*: kg): heart rate (beats/min) = 147.263∙*M* ^-0.141^ + 889.640∙*M* ^-0.179^∙ODBA (*g*). Combining this equation with the previously reported energy expenditure per heartbeat, we estimated the energy expenditure of the tested animals, and the results indicated that ODBA is a good proxy for estimating the energy expenditure of grazing farm animals across species and breeds. The utility and simplicity of the procedure with acceleration loggers could make the accelerometry technique a worthwhile option in field research and commercial farm use.

## Introduction

The efficient utilization of available resources has been of great importance in animal production systems all over the world. In developing countries, extensive pastoral systems tend to occupy the climatologically most unsuitable areas for crop production, and in developed countries, grazing for ruminant animals in pastures and grassland has also become a new trend because of public concerns regarding animal welfare and the environmental impacts of intensive systems [1]. Grassland-ruminant ecosystems are efficient and sustainable methods for converting non-edible resources (grass) into high-protein human foods (i.e., milk and meat). In these ecosystems, grazing animals consume plants growing on grassland, and plant growth is increased by the feces and urine excreted by the animals and deposited and recycled in the field [2]. It is therefore necessary to obtain information about the balance between the grassland production and the energy requirements of grazing animals to realize efficient animal management in grazing systems. In this context, the evaluation of the energy expenditures of farm animals is one of the most important issues in efficient grazing management.

The energy expenditure of animals, known as heat production, has been determined mostly under controlled and confined laboratory conditions in calorimetric or respiratory chambers [3,4]. However, the energy expenditure determined by such methods cannot always be applied to grazing animals because the energy expenditure of grazing animals is greatly influenced by physical activity and various environmental conditions [5]. Accordingly, there is a need for the development of field methods to assess the energy expenditure of grazing animals using techniques that are accurate, noninvasive, inexpensive and robust [6].

There are two major methods for estimating the energy expenditure of animals under unconstrained conditions: the doubly labeled water method and the heart rate method. The doubly labeled water method [7–10] estimates total carbon dioxide production by using an injection of isotope-labeled water, and it then estimates the total energy expenditure from the total carbon dioxide production over the duration of the experiment. The heart rate method [11–13] uses the heart rate as a proxy for the energy expenditure on the basis of the significant relationship between the animals’ heart rates and metabolic rates [14]. When appropriately calibrated, the heart rate method provides an estimate of the rate of oxygen consumption, and consequently energy expenditure, over relatively short as well as over long time periods.

Although these two methods have already been applied to farm animal research, there are some limitations. The doubly labeled water method cannot estimate the energy expenditure for short periods of time (i.e., the possible duration for estimation depends on the animal’s size, ranging from 24 hours for small vertebrates to up to 28 days in humans [15]). Thus, the energetic cost required for specific activities cannot be calculated with this method. In addition, the use of an isotope in the analysis is expensive, especially for large animals. On the other hand, heart rate can be easily influenced by an animal’s affective state/mental stress [16,17], which causes an overestimation of energy expenditure. Further, heart rate loggers are relatively difficult to deploy for free-ranging animals because the loggers’ electrodes must be robustly fixed to, or surgically implanted in, the animal to avoid having the electrodes drop off or the electric cables cut. In particular, for large animals such as cattle and buffaloes, two separate electrodes must be attached at shaved sites using ample electrode gel, and they must be firmly fixed with girth [18]. When implanted electrodes are used, the animals need several days for recovery after the surgery, and as with any invasive procedure, complications can emerge [19]. These techniques are thus not always applicable to the estimation of the energy expenditure of grazing farm animals (see Butler et al. [15] for a detailed discussion), and alternative techniques are required.

An alternative technique to estimate energy expenditure by measuring body acceleration was pioneered by Cavagna et al. [20] and has been widely performed in wildlife and human research fields [21,22]. The concept of this technique is simple: the more actively animals move, the more energy they expend due to the contraction of muscles required for movement. In general, the accelerometry technique can quantify the movement of an animal using acceleration loggers, and the acceleration loggers are relatively easy to deploy due to their miniaturization, which minimizes the physiological and psychological stress to subject animals [23].

In recent years, a new acceleration index named “Overall Dynamic Body Acceleration (ODBA)” [24], which quantifies the three-dimensional movement of animals as the value of acceleration and is assumed to be a proxy for activity-specific energy expenditure, has been used in the field of wild animal ecology. Wilson et al. [24] demonstrated that ODBA was significantly correlated with the rate of oxygen consumption during exercise in great cormorants (*Phalacrocorax carbo*), suggesting the potential of ODBA as a new measurement of the energy expenditure of free-ranging animals. Since then, robust relationships between ODBA and rate of oxygen consumption have been found in a wide range of animals [23,25]. The ODBA method is based on two simple features: (1) it uses the sum of three-dimensional body acceleration and (2) it extracts only dynamic acceleration (due to movement) by subtracting static acceleration (due to gravity) from the raw acceleration data [23,26]. Although ODBA is an easily calculated metric [27], these features raise the accuracy of the ODBA method for predicting energy expenditures [28]. Several studies have used ODBA in field experiments to evaluate energy costs [29] and animal behaviors in response to environmental and physiological conditions [30,31]. Although ODBA has been used to predict activity-specific energy expenditures in a variety of animal species, it has not yet been established whether ODBA can be applied to grazing farm animals, such as cattle, goats and sheep.

The objective of the present study was to test the potential of ODBA as a proxy for the energy expenditure of grazing ruminants by analyzing the effect of ODBA on heart rate, which is a conventional proxy. We measured the ruminants’ body acceleration and heart rate simultaneously on pastures.

## Materials and Methods

### Study sites, periods and tested animals

All field experiments for data collection were conducted in Japan from 2011 to 2013. Three farm animal species (cattle, goats and sheep) were used for the study. The experiments with cattle (*Bos Taurus*) were conducted at two sites: the experiments using three Japanese Black cows were conducted at Ikari Highland Farm (35° 43' N, 135° 12' E), Kyoto, in June and August 2011 and the experiments using five Japanese Brown cows were conducted at Tokai University (32° 53' N, 131° 00' E), Kumamoto, in October 2011, July 2012 and August 2012. The experiments with goats (*Capra hircus*) were conducted at Kyoto University (35° 02' N, 135° 47' E), Kyoto, in June 2011, June 2012 and June 2013, using six castrated Saanen goats. The experiments with sheep (*Ovis aries*) were conducted at Kyoto University in May 2013 and at the University of Shiga Prefecture (35° 15' N, 136° 12' E), Shiga, in June 2013 using five castrated Corriedale sheep.

Table 1 shows the weights and ages of the tested animals and the ambient temperatures during the data collection at each site. The body weights of the animals were measured before the experiments. The ambient temperature (at 10-min intervals) at the study sites was obtained from the Japan Meteorological Agency website [32]. The mean temperatures at all study areas were thermoneutral for the tested ruminants. All tested animals were accustomed to being under grazing management. The animals were given supplemental feed, if necessary, to maintain their weight, and had free access to water. All animal experiments were approved by the Animal Experiment Committees of Kyoto University (Permit Number: 26–76), Tokai University (111102, 122078), and the University of Shiga Prefecture (24–4), and permitted by the farm manager at the Ikari Highland Farm. All of the procedures for equipping the animals with data loggers were performed as quickly as possible to minimize the animals’ discomfort.

**Table 1:** Tested animals and ambient temperature at the study sites.

Species	Breed (Abbr.)	n	Age (months)	Body weight (kg)	Ambient temperature (°C)
Cattle	Japanese Black (JBL)	3	55.3 ± 6.1	488.7 ± 31.7	27.1 ± 2.3
Cattle	Japanese Brown (JBR)	5	146.6 ± 38.5	523.8 ± 40.1	22.8 ± 3.4
Goat	Saanen (SA)	6	67.0 ± 9.8	74.5 ± 7.3	23.7 ± 3.0
Sheep	Corriedale (CO)	5	28.4 ± 1.3	37.1 ± 3.4	21.6 ± 2.2

The age, body weight and ambient temperature data are means ± SD.

### Data collection

**Fig 1:**
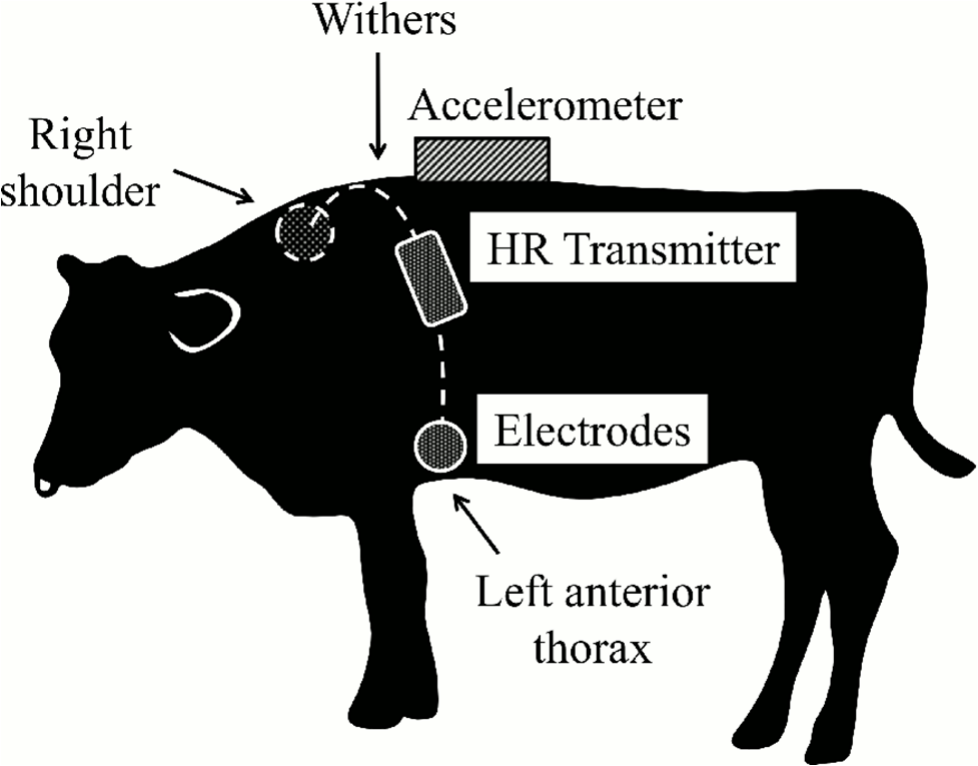
The position of the accelerometer and the electrodes for the heart rate monitor. The position of the accelerometer is at the top of the animal’s back (behind the withers), and the positions of the two electrodes connected to a transmitter of the heart rate monitor are at the animal’s right shoulder and left anterior thorax, which are known to be the appropriate points for heart rate measurements for ruminants.

Acceleration was measured using acceleration data loggers (USB Accelerometer X6-1A, X6-2, Gulf Coast Data Concepts, Waveland, MS, USA) that were set to record the acceleration (±2 g) in three axes separately at 10 Hz with 16-bit resolution. The acceleration data were recorded to a removable microSD card (1 GB). Because ODBA was intended to be a measure of acceleration around an animal's center of mass [23], the acceleration logger was attached at the top of each animal’s back (behind the withers) (Fig 1). Animals are often instrumented with acceleration loggers around their necks [23]. However, the position appears to be unsuitable for ruminants because they move their necks actively when they graze or ruminate, causing additional acceleration independent of movement of the center of an animal’s mass.

The animals’ heart rates were measured using a heart rate monitor (RS800CX, Polar Electro, Kempele, Finland). Electrodes were placed on the animal’s right shoulder and left anterior thorax, fixed to the belt that was fastened securely around the animal’s body. The electrodes were connected to a transmitter of the heart rate monitor with leads for extension because this heart rate monitor was designed for human use (Fig 1). The heart rate monitor with infrared connections to the transmitter recorded the heart rate data at 1-min intervals during the experiment.

For the comparison of the acceleration data and heart rate data to a conventional activity index, we also measured the number of steps (footsteps) for some tested animals using the IceTag data logger (IceTag sensor, IceRobotics, West Lothian, UK). The IceTag sensor measures the intensity of lying, standing, and activity measured as the percent of time spent lying and standing, a motion index, and the number of steps at 1-min intervals [33]. The IceTag sensor was attached at the left hind leg of the tested animals with a Velcro strap.

During the experimental periods, the measurements of body acceleration in three axes, heart rate, and the number of steps were recorded simultaneously. At the end of the experimental period, all loggers were immediately removed, and the data were then downloaded into a personal computer.

### Data processing

The downloaded three-dimensional acceleration data were converted from digital counts into the standard acceleration of gravity (*g*). The raw values of acceleration in each axis were the result of the combination of static acceleration (due to gravity) and dynamic acceleration (due to movement). In the present study, ODBA was derived using the following procedure: (1) static acceleration was first approximated by smoothing the obtained acceleration using 2-sec running means, according to Halsey et al. [25]; (2) dynamic acceleration was calculated by subtracting the static acceleration from the raw acceleration; and (3) ODBA was calculated by summing the three spatial absolute dynamic accelerations. The process of the derivation of ODBA from raw acceleration values in three axes was presented by Gleiss et al. [26]. The mean ODBA per minute determined by averaging the values of ODBA obtained in each minute was used for the analysis. In addition to ODBA, the vector sum of dynamic body acceleration in three dimensions (VeDBA; Qasem et al. [34]) was also calculated to analyze the effect of the differences in dynamic body acceleration indices on the relationship between acceleration and heart rate. Heart rate data were derived from the loggers using Polar Pro Trainer 5 software (Polar Electro). The heart rate data were recorded as beats per minute. To eliminate measurement errors, we removed the values outside the 3-sigma range removed as outliers. The proportion of heart rate data removed was 2.9 ± 1.6% (Mean ± SD). The number of steps during the experiments was also collected from the IceTag sensor using IceTag Analyzer software (IceRobotics).

The measurements of ODBA, VeDBA, heart rate and the number of steps collected in each 1-min interval were averaged over 10-min periods to remove synchronization errors from second-scale time differences among measurements of these indices. Only the data collected with both ODBA and heart rate were analyzed. There were on average 363 pairs of ODBA and heart rate data in each experiment. Each data set corresponded to data acquired over 10 min; therefore, the average period of time recorded was 60.5 h.

### Statistical Analysis

#### The relationship between ODBA and heart rate

We examined the relationship between ODBA and heart rate with general linear models (GLM). Following Halsey et al. [25], the data for all individuals in all species and breeds were included in the following model:

$${\mathrm{HR}}_{ijk}=\mu +G_{i}+A_{j}+\beta_{1}{\left(ODBA\right)}_{ijk}+\beta_{i}{\left(ODBA\right)}_{ijk}+e_{ijk}$$ (1)

where *HR*_*ijk*_ is heart rate (beats/min), *μ* is the overall mean, G_*i*_ is the random effect of the group of species and breeds (Japanese Black cattle: JBL, Japanese Brown cattle: JBR, Saanen goat: SA, and Corriedale sheep: CO), *A*_*j*_ is the random effect of individual animals nested within the group, *β*_*1*_ *(ODBA)*_*ijk*_ is the covariate of ODBA (*g*), *β*_*i*_ *(ODBA)*_*ijk*_ is the interaction between ODBA and the group of species and breeds (*β*_*1*_ is the overall regression coefficient and *β*_*i*_ *is* the effect of the *i*th group to the regression coefficient) and *e*_*ijk*_ is the residual error. The effect of individuals on the relationships between ODBA and heart rate in each group of species and breeds was also analyzed with the following model:

$$HR_{ij}=\mu +A_{i}+\beta_{1}{\left(ODBA\right)}_{ij}+\beta_{i}{\left(ODBA\right)}_{ij}+e_{ij}$$ (2)

where *HR*_*ij*_ is heart rate (beats/min), *μ* is the overall mean, *A*_*i*_ is the random effect of the individual, *β*_*1*_ *(ODBA)*_*ij*_ is the covariate of ODBA (*g*), *β*_*i*_ *(ODBA)*_*ij*_ is the interaction between ODBA and the individual (*β*_*1*_ is the overall regression coefficient and *β*_*i*_ *is* the effect of the *i*th group to the regression coefficient), and *e*_*ij*_ is the residual error. The interaction terms in models (1) and (2) tested whether the relationship between ODBA and heart rate varied significantly among the groups and individuals, respectively. In addition to ODBA, the effect of the two other activity indices, the number of steps and the VeDBA, on heart rate was also analyzed by replacing the covariate of ODBA into the indices in models (1) and (2), respectively. Moreover, to verify the effect of the differences in body mass of animals on the relationship, the effects of species and breeds on the intercept and slope of the regression equation between ODBA and heart rate for each individual were investigated by GLM, including the effect of the group, and the differences were analyzed using the least squares means with the Tukey–Kramer adjustment.

#### Estimation for the energy expenditure from ODBA across different species and breeds

**Fig 2:**
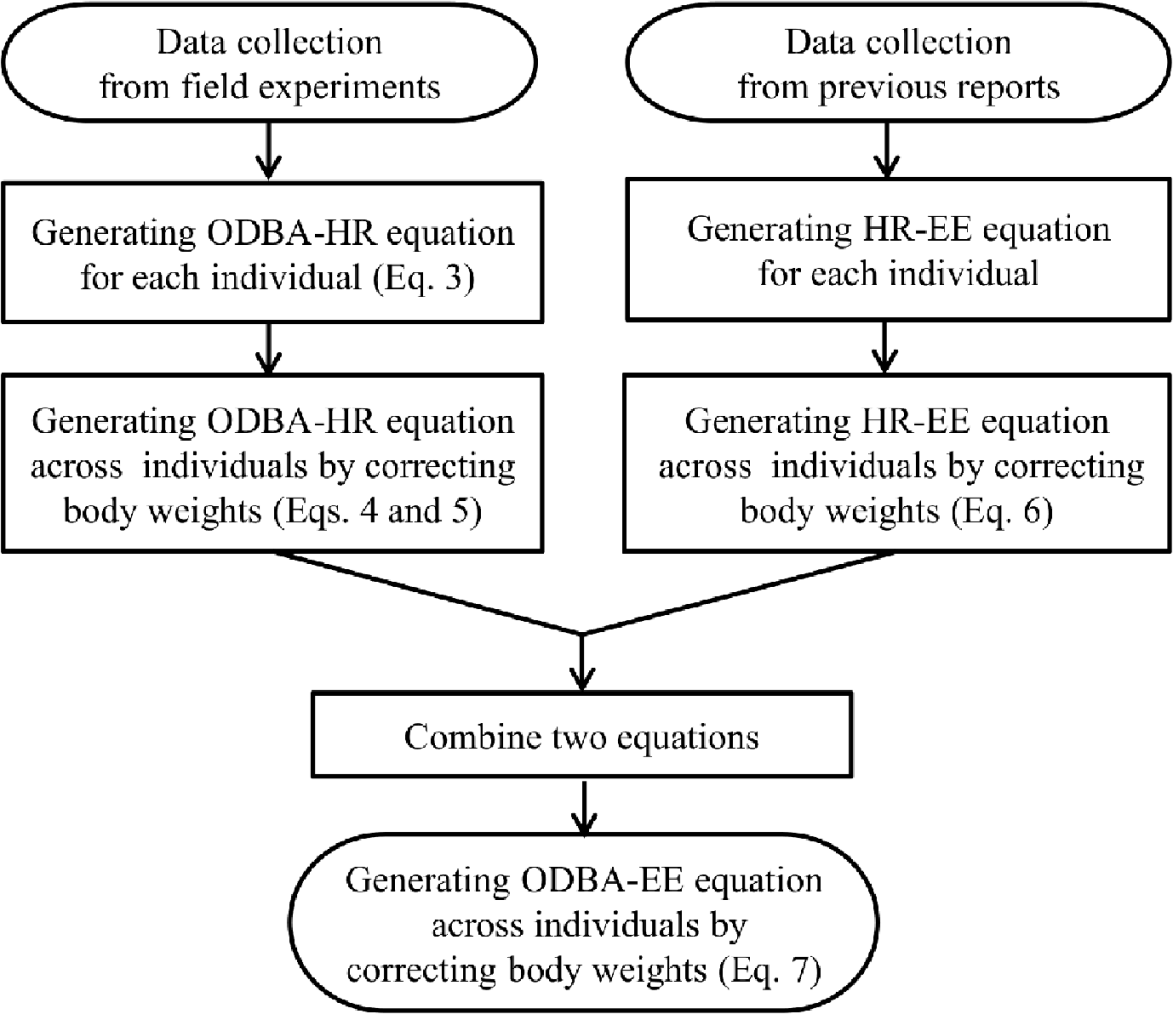
Flowchart of the data analysis. The equation numbers in the figures correspond to those in the text.

Following the procedure for generating the equation to estimate the energy expenditure from ODBA for the tested animals (Fig 2), we first examined the relationship between ODBA and heart rate for each animal to estimate the energy expenditure across different species and breeds. A simple regression analysis of the relationship between ODBA and heart rate was executed as:

$$HR=b_{0}+b_{1}\cdot ODBA$$ (3)

where *HR* is heart rate (beats/min), *ODBA* is overall dynamic body acceleration (*g*), and *b*_*0*_ and *b*_*1*_ are the intercept and slope of the regression equation, respectively.

Second, the effects of body mass on the intercept and slope of the regression equations were analyzed by allometric equations:

$$b_{0}=ci\cdot M^{di}$$ (4)

$$b_{1}=cs\cdot M^{ds}$$ (5)

where *b*_*0*_ and *b*_*1*_ are the intercept and slope of the regression equation between ODBA and heart rate for each animal, respectively, and *M* is the body weight of each animal (kg). The coefficients of *ci* and *di* for the intercept and *cs* and *ds* for the slope were estimated after the transformation of Eqs 4 and 5 to the natural logarithm function as *logb*_o_ *= logci +di∙logM* and *logb*_1_ *= logcs +ds∙logM*, respectively.

Third, the energy expenditure was estimated from ODBA using the equations derived from the relationships between the heart rate and energy expenditure for the same species and breeds in previous Japanese experimental studies [35–37]. The relationships between heart rate and energy expenditure for the same species and breeds were meta-analyzed by the following allometric equation:

$$eEE=f\cdot HR^{g}\cdot M^{h}$$ (6)

where e*EE* is the estimated energy expenditure (kJ/h) and *HR* is heart rate (beats/min). The coefficients of *f*, *g*, and *h* were estimated after the transformation of Eq 6 to the natural logarithm function as *logeEE = logf +g∙logHR+h∙logM*. Combining this estimated equation (Eq 6) with the estimated intercept and slope of the regression equation between the ODBA and heart rate obtained in the present study (Eq 4 and Eq 5, respectively), the estimated energy expenditure for the tested animals was calculated using ODBA by the following equation:

$$eEE=f\cdot {\left(ci\cdot M^{di}+cs\cdot M^{ds}\cdot ODBA\right)}^{g}\cdot M^{h}$$ (7)

where the content in the parenthesis indicates the regression equation between ODBA and heart rate.

All statistical analyses were performed with SAS (SAS Institute, Cary, NC) [38].

## Results

### The relationship between ODBA and heart rate

**Fig 3:**
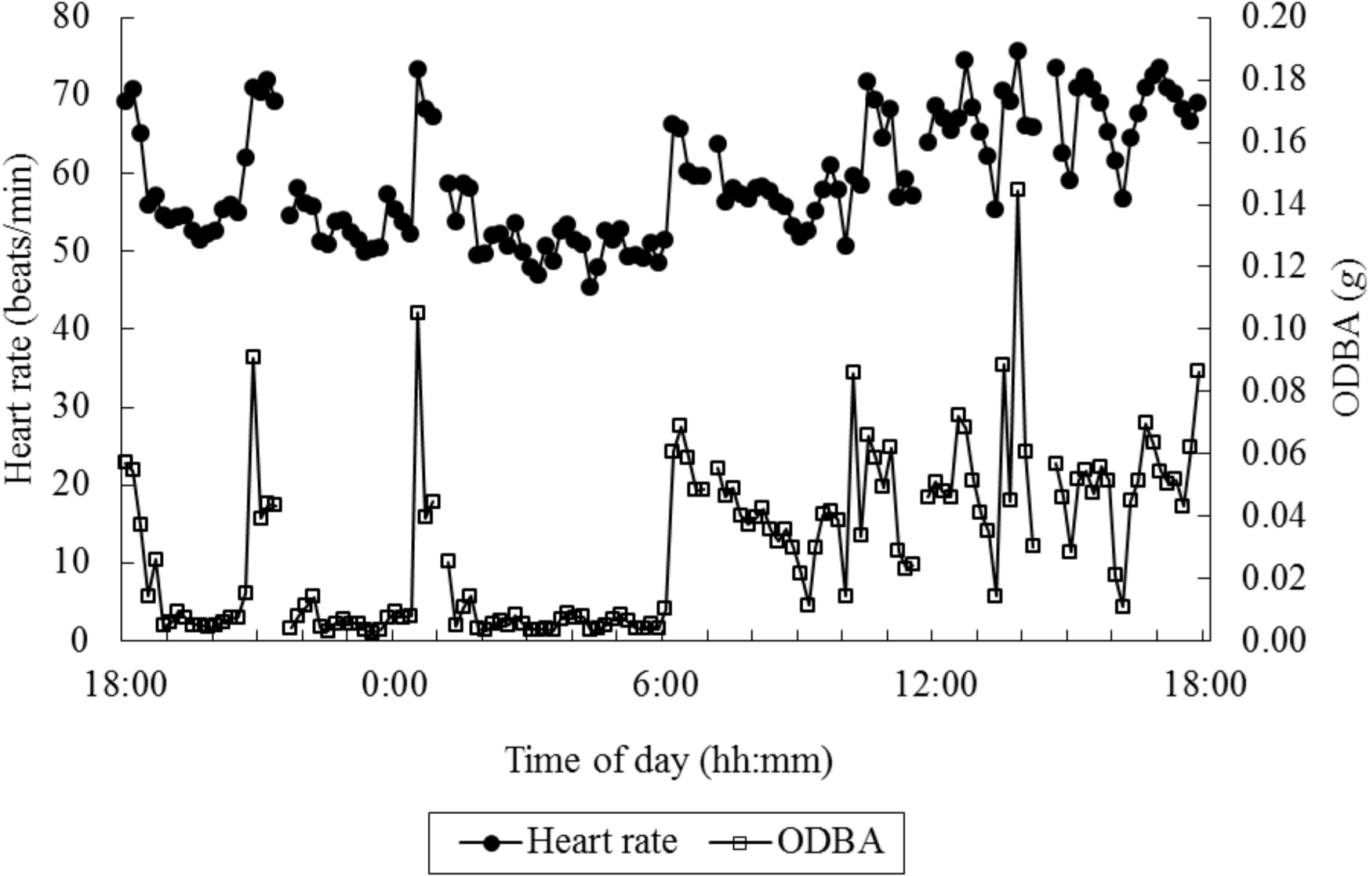
An example data plot of overall dynamic body acceleration (ODBA) and heart rate per minute over a 24-h recording. The interruptions in the recording indicate the temporal interruptions of the heart rate recording, whereas ODBA was recorded continuously throughout the experiment. The interruptions in ODBA occurred due to the synchronization of the heart rate and ODBA for the data analysis.

The coefficients of determination (*R* ^*2*^) obtained from GLM for heart rate in all data and in each group when ODBA, the number of steps or VeDBA was used as the covariates are shown in Table 2. In all models, the effects of activity indices on heart rate were significant (*P*<0.01), indicating that all three activity indices were significantly related with heart rate. The *R* ^*2*^ of the GLM with ODBA was 0.868, which was higher than the *R* ^*2*^ with the number of steps (0.799) and was slightly higher than the *R* ^*2*^ with VeDBA (0.866). In the models by each group, the mean *R* ^*2*^ of the GLM with ODBA was also higher than those with the number of steps or VeDBA (ODBA: 0.730 ± 0.031, the number of steps: 0.507 ± 0.123, VeDBA: 0.728 ± 0.031). These results indicated that ODBA was the most accurate predictor for heart rate in the present study. Fig 3 shows a plot of the ODBA and heart rate for a typical 24-h period extracted from one animal experiment. Although the fluctuation was large within the day, the general patterns of change in ODBA and heart rate were similar.

**Table 2:** The coefficients of determination (*R* ^*2*^) by GLM analysis for all data and for each group when the effects of activity indices on heart rate were analyzed.

Model	*R* ^*2*^		
	ODBA*	The number of steps	VeDBA*
All animals**	0.868	0.799	0.866
Group of species and breeds***			
Japanese Black cow	0.690	0.472	0.688
Japanese Brown cow	0.752	0.406	0.753
Saanen goat	0.757	0.645	0.753
Corriedale sheep	0.721	n.d.****	0.719

All factors and interactions were significant (*P*<0.01).

* ODBA and VeDBA are overall and vectorial dynamic body accelerations (*g*), respectively.

** The model for all individuals has the form *HR*_*ijk*_ = *μ + G*_*i*_ + *A*_*j*_ + *β*_*1*_ *(ODBA)*_*ijk*_ + *β*_*i*_ *(ODBA)*_*ijk*_ + *e*_*ijk*_, where *HR*_*ijk*_ is heart rate (beats/min), *μ* is the overall mean, G_*i*_ is the random effect of the group of species and breeds (Japanese Black cattle: JBL, Japanese Brown cattle: JBR, Saanen goat: SA, and Corriedale sheep: CO), *A*_*j*_ is the random effect of individual animals nested within the group, *β*_*1*_ *(ODBA)*_*ijk*_ is the covariate of ODBA (*g*), *β*_*i*_ *(ODBA)*_*ijk*_ is the interaction between ODBA and the group of species and breeds (*β*_*1*_ is the overall regression coefficient and *β*_*i*_ *is* the effect of the *i*th group to the regression coefficient) and *e*_*ijk*_ is the residual error.

*** The model for each group of species and breeds has the form *HR*_*ij*_ = *μ + A*_*i*_ +*β*_*1*_ *(ODBA)*_*ij*_ + *β*_*i*_ *(ODBA)*_*ij*_ + *e*_*ij*_, where *HR*_*ij*_ is heart rate (beats/min), *μ* is the overall mean, *A*_*i*_ is the random effect of the individual, *β*_*1*_ *(ODBA)*_*ij*_ is the covariate of ODBA (*g*), *β*_*i*_ *(ODBA)*_*ij*_ is the interaction between ODBA and the individual (*β*_*1*_ is the overall regression coefficient and *β*_*i*_ *is* the effect of the *i*th group to the regression coefficient), and *e*_*ij*_ is the residual error.

**** n.d.: No data.

With regard to other factors in the models, all factors and interactions were significant in the three models for all data (*P*<0.01), indicating that there were variations in the relationship between activity indices and heart rate among groups of species and breeds. When the effect of individuals on the relationship between the activity index and heart rate was analyzed for each group, all factors and interactions in all cases were also significant (*P*<0.01), which indicated that there were different effects of individuals on heart rate in each group.

### Estimation of energy expenditure from ODBA

**Fig 4:**
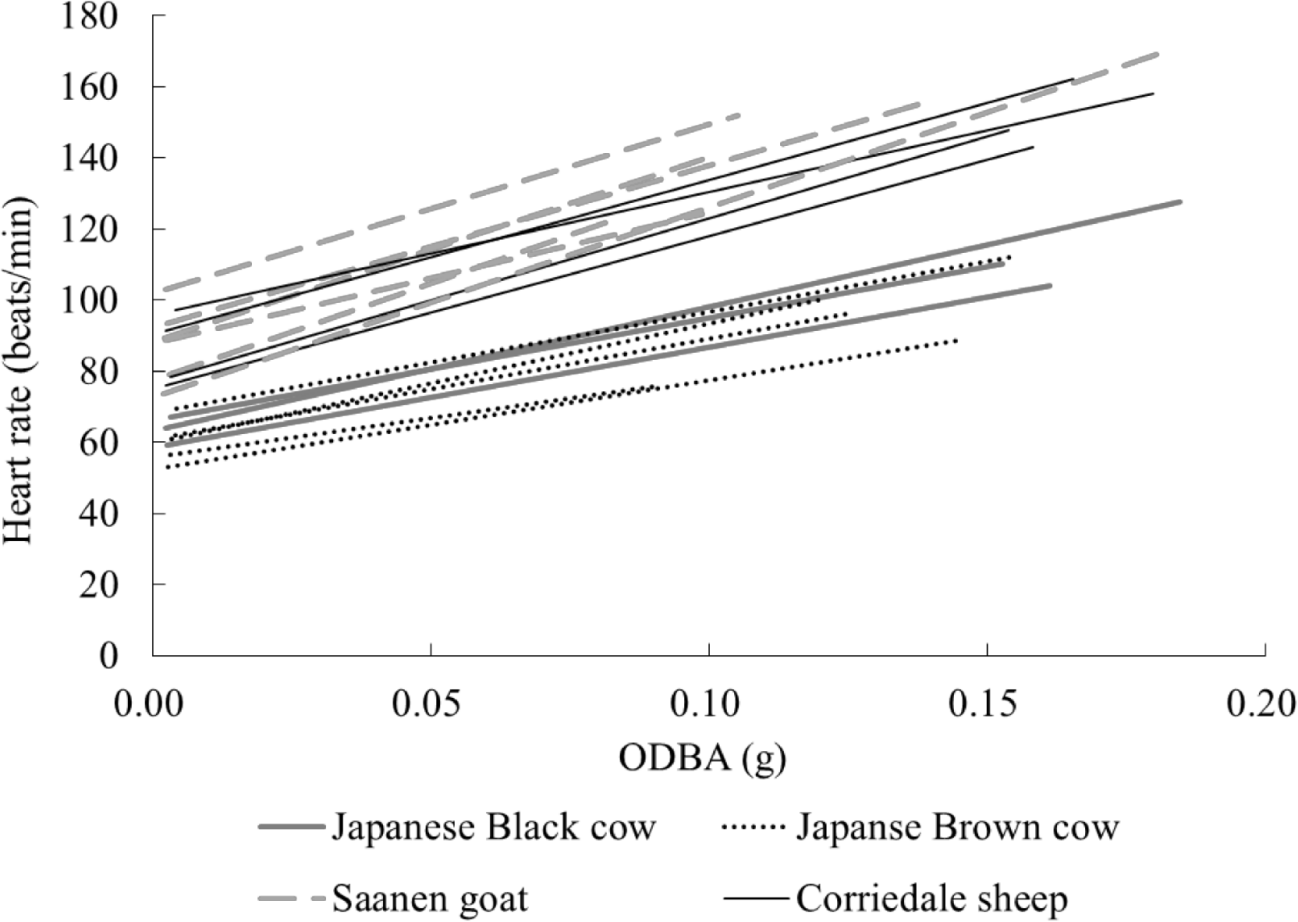
The linear regressions between overall dynamic body acceleration (ODBA) and heart rate for all tested animals. The lines with the same colors correspond to those in the same groups of species and breeds.

The linear regressions between ODBA and heart rate for all tested animals are shown in Fig 4, and the intercepts and slopes for the animal groups are presented in Table 3. There were significant effects of the groups of species and breeds on both the intercept and slope of the regression equations (*P*<0.01). However, the intercepts were significantly different between only the small (Saanen goats and Corriedale sheep) and large ruminants (Japanese Black cows and Japanese Brown cows) (*P*<0.05). The slopes for the Corriedale sheep and Japanese Black cows tended to be different (*P* = 0.07), indicating that the slopes were also different between small and large ruminants.

**Table 3:** Multiple comparisons of the intercept and slope of linear regression equations between overall dynamic body acceleration (ODBA) and heart rate in the species and breeds tested.

Animal	Intercept (*b*_*0*_)*	Slope (*b*_*1*_)*
Japanese Black cow	62.61 ± 4.87^a^	306.90 ± 30.06^a^ ^b^
Japanese Brown cow	59.52 ± 3.77^a^	274.77 ± 23.29^a^
Saanen goat	86.81 ± 3.44^b^	483.09 ± 21.26^c^
Corriedale sheep	83.42 ± 3.77^b^	408.78 ± 23.29^b^ ^c^

The effects of the groups of species and breeds on the intercept and slope were significant (*P*<0.01). The linear relationship has the form *HR = b*_*0*_ *+ b*_*1*_ *∙ODBA*, where *HR* is heart rate (beats/min), *ODBA* is overall dynamic body acceleration (*g*), and *b*_*0*_ and *b*_*1*_ are the intercept and slope of the regression equation, respectively.

*Values are least square means with standard errors.

^a,b,c^ Values within a column with different superscripts differ significantly (*P*<0.05).

The results of the allometric fittings for the intercept and slope of the linear regression equations relative to body weight (Eqs 4 and 5) showed that the relationships between individual body weights (*M*, kg) and both the intercept and slope of the linear regression equation for all tested animals were significant (*P*<0.01). The intercept and slope values were *b*_0_ = 147.263∙*M* ^−0.141^ (*R* ^*2*^ = 0.674) and *b*_1_ = 889.640∙*M* ^−0.179^ (*R* ^*2*^ = 0.577), respectively. Thus, the resultant equation for estimating heart rate (*HR*, beats/min) from ODBA (*ODBA*, *g*) across species and breeds by correcting differed body weights was as follows:

$$HR=147.263\cdot M^{-0.141}+889.640\cdot M^{-0.179}\cdot ODBA.$$ (8)

In the present study, we estimated the energy expenditure from ODBA using the relationship between heart rate and energy expenditure, which was estimated from the regression analysis of the data reported in ruminants [35–37] using an allometric equation (Eq 6). The resultant equation was:

$$eEE=2.907\cdot HR^{0.516}\cdot M^{0.777}$$ (9)

where e*EE* is the estimated energy expenditure (kJ/h) (n = 23, *R* ^*2*^ = 0.996).

Combining Eq 8 with Eq 9, the equation for estimating the energy expenditure directly from ODBA was obtained:

$$eEE=2.907\cdot {\left(147.263\cdot M^{-0.141}+889.640\cdot M^{-0.179}\cdot ODBA\right)}^{0.516}\cdot M^{0.777}.$$ (10)

When this equation is applied for the data obtained in the present study, the mean energy expenditure of grazing ruminants was estimated as 752.0 ± 4.2 kJ/M^0.75^∙day in Japanese Black cows, 735.3 ± 6.7 kJ/M^0.75^∙day in Japanese Brown cows, 806.4 ±5.6 kJ/M^0.75^∙day in Saanen goats, and 854.5 ± 7.6 kJ/M^0.75^∙day in Corriedale sheep.

## Discussion

### The technical usability of the acceleration technique

It should be noted that in the present study, the acceleration data could be recorded continuously throughout all of the experiments, whereas an interruption in the heart rate recording was observed in six experiments (Fig 3). This revealed the ease of collecting acceleration data for animals at pasture compared to heart rate data; the heart rate measurements required the placement of electrodes on the appropriate area of each animal’s body, and under grazing management, animals are so active that electrodes can often be detached and easily lost [15]. Thus, it is difficult to record the heart rate of free-ranging animals for a long period without the surgical implantation of electrodes, which is a substantial disadvantage of this method. In contrast, acceleration loggers are very small and are relatively easy to deploy and to maintain reasonably firmly against an animal’s body using a strap, belt or harness [23]. As long as an acceleration logger is fixed on a grazing animal’s body, it can record the animal’s acceleration data over a long period. Therefore, even if the acclerometry technique has comparative or less prediction accuracy in estimating energy expenditures to that of the heart rate method, it could be a worthwhile option in terms of convenience in field research and also in commercial farm use.

### The comparison of ODBA to the number of steps and VeDBA

The energy expenditures of grazing animals appear to be considerably higher than those of housed animals, due mainly to increases in walking distance and other activities for grazing animals [39]. The activity indices should thus be incorporated into the model for estimating the energy expenditure of free-ranging animals. The number of steps has been conventionally used to quantify the animal’s activity [40,41]. Shibata et al. [40] demonstrated, in a laboratory experiment using a treadmill, that the number of steps had a positive correlation with the energy expenditure of dairy heifers, suggesting the potential of the measurement of the number of steps for estimating the energy expenditures of animals during grazing. In the present study, however, the *R* ^*2*^ values obtained from the GLM of the relationship between the number of steps and heart rate were lower than those between ODBA and heart rate (Table 2). This suggested that ODBA can be a better activity index to estimate heart rate and consequently energy expenditure for individual animals than the number of steps.

The difference between the results of ODBA and the number of steps may be explained by the effect of the terrain. In previous studies, the energy expenditure during uphill running increased linearly with the angle of the incline, whereas the energy expenditure during moderate downhill running decreased [42,43]. The method to estimate the energy expenditure by counting the number of steps entails the assumption that the energy cost per step is constant throughout grazing, but this assumption is not realistic because the energy cost per step could vary widely depending on the slope gradient and the body size of animals [44]. In contrast, the body acceleration for animals moving up a slope can be different depending on the slope gradient. Herren et al. [45] reported that the vertical acceleration of human running increases as the gradient increases, which indicates that the tri-axial acceleration index can quantify the vertical movement of an animal walking on a gradient and that this could therefore explain the effect of the terrain on the cost of the locomotion activity. The results of the present study may highlight the value of ODBA in being able to allude to proper power terms rather than simply counting steps; although both indices quantify body movements of animals, the values of ODBA will rise with both increasing stride frequency and amplitude, whereas step counters can only rise with frequency. Moreover, although locomotion activities are predominant factors contributing to increases in the energy expenditures of grazing animals, other activities, such as insect avoidance and standing-up/lying-down behavior, also contribute to the increase in the energy expenditure. An acceleration index, such as ODBA, can quantify such activities and thereby account for the additional energy expenditure for the activities, whereas the number of steps can reflect only the locomotion activities.

The results for ODBA and VeDBA showed that both indices were significantly related with heart rate and that the *R* ^*2*^ values obtained from the GLM were slightly higher by ODBA than by VeDBA, which was in accordance with Qasem et al. [34] who reported that ODBA appeared to be a marginally better proxy for rate of oxygen consumption (VO_2_) than VeDBA. Gleiss et al. [26] noted that the ODBA method required the orientation of the device to be consistent between individuals (each data collection) because the values of ODBA obtained will change according to the device orientation between individuals. For arbitrary orientations with respect to gravity in particular, ODBA can both exaggerate and underestimate changes in the acceleration magnitude caused by rotation [46]. In the present study, because the instrumented position of the accelerometers (behind the withers of animals) was standardized through all data collection, the estimation error in ODBA due to the variation of the device orientation might be minimized. However, in the situation where consistent device orientation is not guaranteed, the use of VeDBA can be effective. In fact, in the present study, the differences in *R* ^*2*^ values from the GLMs using ODBA and VeDBA were small, indicating that VeDBA is also a good proxy for estimating energy expenditure. As a reference, the equation for estimating the energy expenditure (*eEE*, kJ/h) directly from VeDBA (*VeDBA*, *g*) across species and breeds by correcting differed body weights was also established as:

$$eEE=2.907\cdot {\left(147.480\cdot M^{-0.141}+1262.810\cdot M^{-0.172}\cdot VeDBA\right)}^{0.516}\cdot M^{0.777}$$ (11)

where the content in the parenthesis indicates the regression equation between VeDBA and heart rate (Intercept: *R* ^*2*^ = 0.675, Slope *R* ^*2*^ = 0.556). The intercept in the parenthesis is almost identical to that in the equation for ODBA (Eq 10) and the slope for VeDBA (1262.810∙*M* ^−0.172^) divided by that for ODBA (889.640∙*M* ^*-*0.179^ in Eq 10) ranges from 1.45 to 1.48 when the body weight increases from 30 to 600 kg, which might reflect the relationship between VeDBA and ODBA (VeDBA ≈ 2/3ODBA) reported by Spivey and Bishop [46].

### The relationship between ODBA and heart rate across grazing ruminants

We investigated the relationship between ODBA and heart rate for grazing ruminants in the present study. The results revealed significant relationships between ODBA and heart rate. Given that heart rate is assumed to be a reliable proxy for energy expenditure, ODBA could potentially be a good proxy for the energy expenditure of grazing ruminants, as was previously reported for other species [23,25].

The relationship between ODBA and heart rate showed significant differences among groups of species and breeds and also among individuals (*P*<0.01) (Table 2), suggesting the existence of differing relationships among the groups of species and breeds and individuals. Halsey et al. [25] investigated the relationship between ODBA and rate of oxygen consumption in various species and concluded that these relationships varied among the types of animals studied, ranging from bipeds (several bird species to humans) to quadrupeds (several mammal species, such as armadillo, coypu and skunk). However, the difference in the regression equation among the groups of species and breeds and among individuals in the present study could be partly explained by body weight. The intercepts of the regression equation were significantly different (*P*<0.05) and the slopes also tended to be different *(P* = 0.07) between the small ruminants (Saanen goats and Corriedale sheep) and the large ruminants (Japanese Black cows and Japanese Brown cows) (Table 3). This result indicated the effect of body weight on the regression equation between ODBA and heart rate. In addition, following Halsey et al. [25], when we used allometric equations to examine whether the animals’ body weights affected the relationship between ODBA and heart rate across the groups of species and breeds, the results showed that the effects of body weight on both the intercept and slope of the linear relationships for all species and breeds were significant (*P*<0.01).

In the resultant equation (Eq 8), the intercept and the slope can be regarded as the elements that explain the energy expenditure under the stationary (or inactive) and active state, respectively. In addition, the mass exponent of both the intercept and slope (*di* in Eq 4 and *ds* in Eq 5) were less than 1.0, indicating that the energy expenditure per kg body weight tended to be lower in larger species under both the stationary and active state. This result indicates that on a mass-specific basis, the larger species are more efficient in their use of energy, which is in agreement with previous reports [25, 47–49].

Gleiss et al. [26] noted that, by correcting the difference in body weight, the relationship between ODBA and the rate of oxygen consumption may be similar for animals that have similar body structures and use the same propulsive modes. This indicated the possibility of establishing a common prediction equation for energy expenditure from ODBA across species. In fact, Halsey et al. [25] established the prediction equation for the rate of oxygen consumption from ODBA across a range of bipedal and quadrupedal species, but the equation did not include the data for ruminants. The animal species tested in the present study—cattle, goats and sheep-have a similar locomotion mode, and thus the relationship between the ODBA and heart rate of these ruminants was similar after correcting for body weight (Eq 8). Therefore, we have actually established a common prediction equation for energy expenditure from ODBA, which is applicable to ruminants of known body mass.

### Indirect estimation of the energy expenditure from ODBA

Several studies have evaluated the energy expenditure of grazing ruminants. For example, the Commonwealth Scientific and Industrial Research Organization [50] stated that it was assumed that grazing activity increased the energy requirements of dairy cattle relative to maintenance by 20% on flat terrain and by as much as 50% on a hilly pasture. Osuji [51] also noted that cows at pasture would have maintenance needs 50% to 100% greater than similar housed cows.

**Fig 5:**
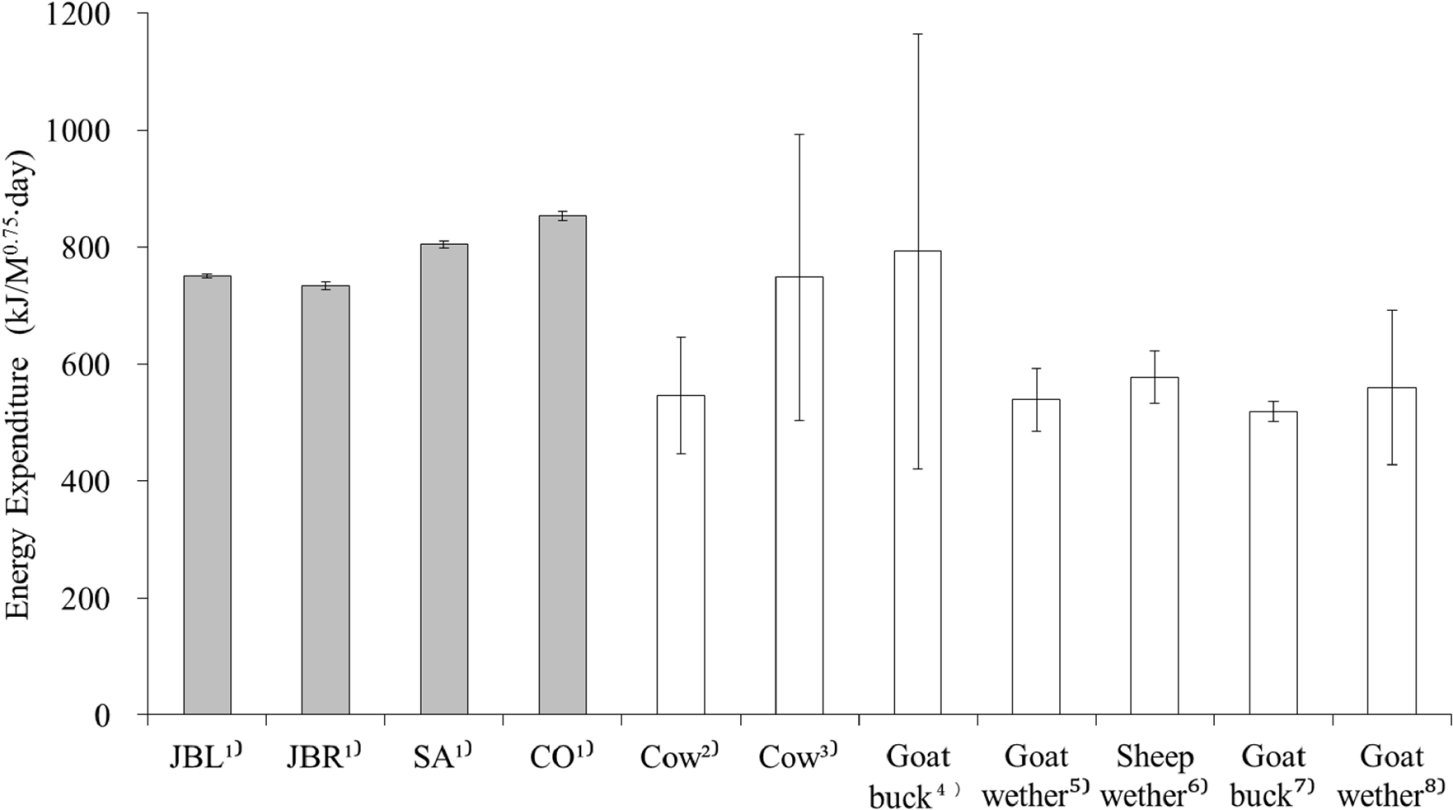
The energy expenditure of grazing ruminants estimated in the present study and in previous reports. 1) (Gray bars) The estimated energy expenditure of Japanese Black cow (JBL), Japanese Brown cow (JBR), Saanen goat (SA) and Corriedale sheep (CO) with accelerometry in the present study (in combination with the relationship between heart rate and energy expenditure derived from the previous reports [35–37]); 2) and 3) The whole energy cost of grazing cows estimated from the heart rate in combination with oxygen consumption per heart beat (O_2_ pulse) by Aharoni [41] and Brosh et al. [52], respectively; 4) The estimated energy expenditure of grazing goats during different seasons (winter, summer and monsoon) in India by collecting the expired air in short periods (5–10 min), reported by Shinde et al. [53]; 5) and 6) The estimated energy expenditure of grazing sheep and goats at different stocking rates from heart rate measurements with O_2_ pulse by Animut et al. [54], respectively; and 7) and 8) The estimated energy expenditure of goat bucks and wethers in open range by the doubly labeled water method by Toerien et al. [9], respectively. The low standard deviations in the present study might be attributed to the condition of experiments (i.e., the use of one breed at a similar stocking rate under thermoneutral conditions) in each animal group.

In the present study, the energy expenditure of grazing ruminants could not be estimated from ODBA directly because a calibration experiment was not conducted. As an alternative, we estimated the energy expenditure from ODBA indirectly in combination with previous data [35–37] (Eq 10). Compared with reported values estimated by other techniques [9,41,52–54], our estimated values of the energy expenditure were slightly higher or within the appropriate range (Fig 5). Some of these differences in energy expenditures may be attributed to differences in activity among species and breeds, conditions of the pastures, the hours of activity, the horizontal and vertical walking distances and variations in inter-individual interactions caused by different stocking densities in various studies [52,54].

It should be noted that the energy expenditure estimated in the present study might involve estimation errors because of the indirect estimation using Eq 9 (the regression equation for estimating energy expenditure from heart rate); when heart rate (beats/minute) was assumed to be 60, the energy expenditure per metabolic body weight estimated from Eq 9(665 kJ/M^0.75^) was higher than those of previous reports (e.g., 414 kJ/M^0.75^ for grazing beef cow in Brosh [12]; 354 kJ/M^0.75^ for sheep and goats in Animut et al. [54]). To improve the estimation and move toward the practical use of the ODBA method, the regression equation (Eq 9) should be improved by collecting more variety of individuals (e.g., sex, age, body mass and nutritional state). Moreover, the direct calibration of the relationship between ODBA and rate of oxygen consumption of ruminants in a respirometry chamber equipped with a treadmill (e.g., Halsey et al. [55]) will be required, and the general validation of the ODBA method in pastures using concomitant techniques, such as the doubly labeled water method, might be informative [25]. Nevertheless, the results of the present study demonstrated that the ODBA method undoubtedly has great potential in estimating the energy expenditure of grazing farm animals.

### A comparison of energy expenditure estimations among diverse animal species

**Fig 6:**
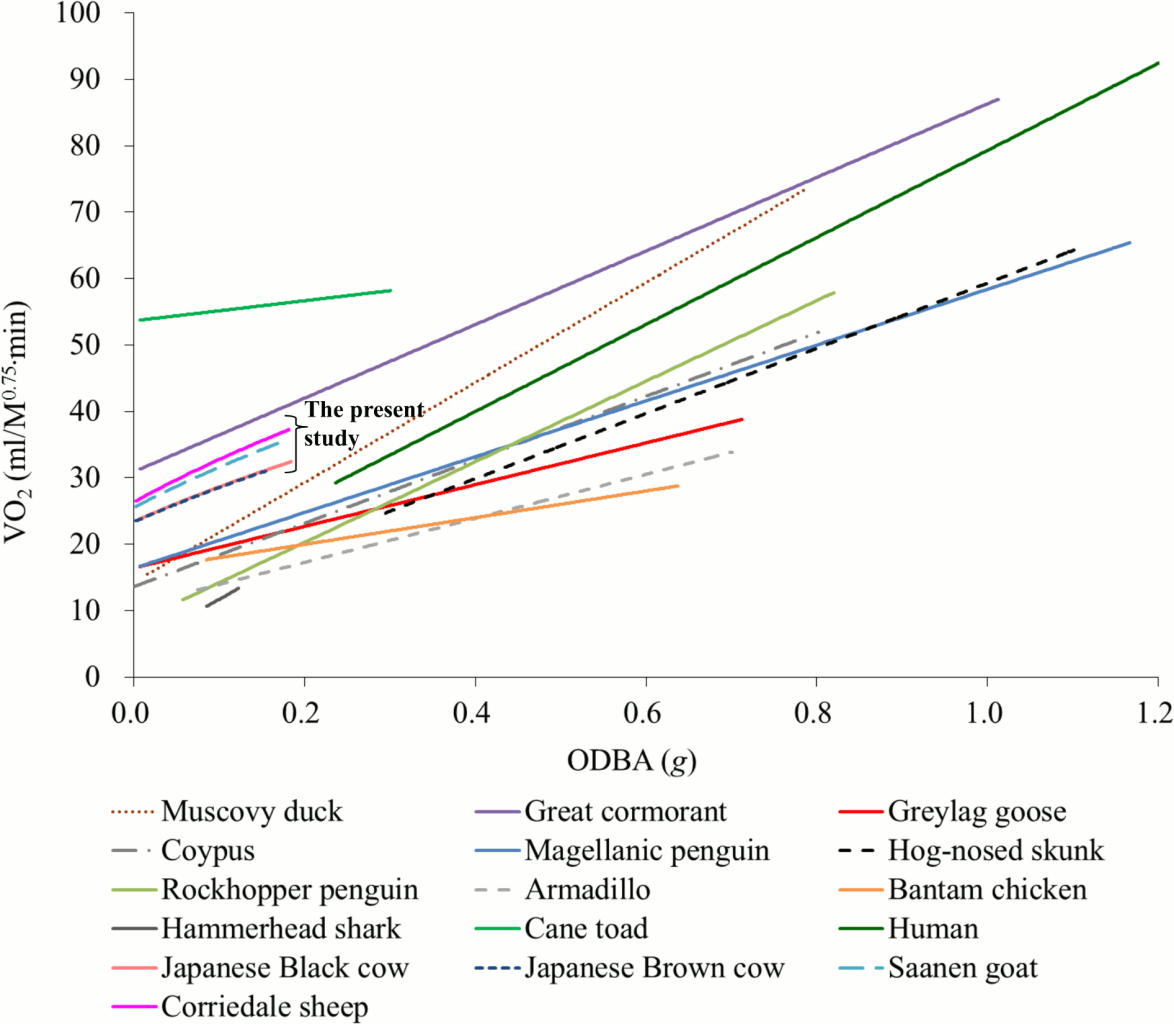
Relationships between the rate of oxygen consumption and overall dynamic body acceleration (ODBA) for a range of bipedal and quadrupedal species. Data other than those for ruminants in the present study were obtained from Halsey et al. [23]. To compare the rate of oxygen consumption (VO_2_, ml/min) between a wide range of animals with different body weights, VO_2_ (ml/min) was converted to VO_2_ per metabolic body weight (ml/ *M* ^0.75^∙min). The VO_2_ of the animals in the present study was estimated by converting *eEE* derived from Eq 10 into VO_2_ using the heat production per unit of VO_2_ for ruminants (20.46 J/ml of VO_2_) given by McLean [56]. The lines for Japanese Black cows and Japanese Brown cows seem to overlap because the body weights were almost identical for these two animals. The line for humans is presented only in the same range of ODBA for the other species.

Finally, we compared the relationships between ODBA and energy expenditures of the tested animals in the present study with those of 12 animal species reported by Halsey et al. [23] (Fig 6). The energy expenditure was expressed by *eEE* (kJ/h) from Eq 10 in the present study and by VO_2_ (ml/min) in Halsey et al. [23]; therefore, we converted *eEE* to VO_2_ using the heat production per unit of VO_2_ for ruminants (20.46 J/ml of VO_2_) given by McLean [56]. Although the Y-axis of the figure in Halsey et al. [23] was expressed by VO_2_ (ml/min), we set the unit of the Y-axis as VO_2_ (ml/min) per metabolic body weight (*M* ^0.75^) to adjust for considerably large body weight differences among animal species. From the comparisons, it could be concluded that the relationships between ODBA and energy expenditures are comparable among several animal species after corrections for body mass.

When the linear relationships between ODBA and VO_2_ for the grazing ruminants were re-estimated by the ODBA-VO_2_ non-linear relationship in Eq 10, the common equation for estimating VO_2_ (*VO*_*2*_, ml/min) from ODBA (*ODBA*, *g*) among diverse animal species including the results by Halsey et al. [23] could be established by correcting differed body weights (Intercept: *R* ^*2*^ = 0.908, Slope *R* ^*2*^ = 0.959) as:

$$VO_{2}=15.139\cdot M^{0.794}+38.467\cdot M^{0.835}\cdot ODBA.$$ (12)

Although the method proposed in the present study is an indirect method for estimating energy expenditure from ODBA, the results of the present study demonstrated that the method might have the potential to estimate the energy expenditure of various types of animal species as well as farm ruminant animals.

## Conclusions

Understanding physiological and behavioral changes of grazing farm animals in comparison with housed farm animals is essential for efficient animal management. Estimating the energy expenditure of farm animals at pasture is especially important, but the presently available methods are limited. Here, we tested the potential of an accelerometry technique for estimating the energy expenditure of grazing ruminants through evaluations of the relationship of an acceleration index, ODBA, with heart rate (a conventional proxy for estimating energy expenditures). The results showed the significant effect of ODBA on heart rate, indicating the possibility that ODBA would be a good proxy for estimating the energy expenditure of grazing farm animals. The other activity indices (the number of steps and VeDBA) also significantly affected heart rate, but ODBA was the highest predictor for heart rate. Finally, by standardizing the placement of accelerometers for all tested animals, a general relationship between ODBA and energy expenditure for farm ruminant species could be derived after correcting for body mass in the present study.

The utility and simplicity of acceleration loggers could enable the wide use of the ODBA method as a better option for estimations of the energy expenditures of pastured animals, especially in field research and commercial farm use. The ODBA method could contribute to knowledge of the energy expenditures of grazing farm animals, and the information gained could be returned to farmers in the form of reports on energy requirements in several feeding standards. Furthermore, the comparison of the energy expenditure estimations from ODBA among diverse animal species in the present study suggested that the relationships would be comparable among several animal species after correction for body mass. Although there might not be a way to standardize the placement between different animal types (bipeds, quadrupeds, aquatic, terrestrial, etc.), at least within groups of the animal types (i.e., quadrupeds in the present study), provided the placement is comparable, there appears to be great potential for deriving the relationships beyond animal species.

## Supporting Information

S1 File The data used in the present study. (XLSX)

## References

[1] Lachica M, Aguilera JF. Estimation of energy needs in the free-ranging goat with particular reference to the assessment of its energy expenditure by the ^13^C-bicarbonate method. Small Rumin Res. 2003; 49: 303–318.

[2] Tilman D, Cassman KG, Matson PA, Naylor R, Polasky S. Agricultural sustainability and intensive production practices. Nature. 2002; 418: 671–677. 12167873 10.1038/nature01014

[3] Frappell PB, Blevin HA, Baudinette RV. Understanding respirometry chambers: what goes in must come out. J Theor Biol. 1989; 138: 479–494. 2593683 10.1016/s0022-5193(89)80046-3

[4] Koteja P. Measuring energy metabolism with open-flow respirometric systems: which design to choose?. Funct Ecol. 1996; 10: 675–677.

[5] National Research Council. Nutrient requirements of Goats Angora, dairy, and meat goats in temperate and tropical countries. Washington: National Academy Press; 1981.

[6] Puchala R, Tovar-Luna I, Goetsch AL, Sahlu T, Carstens GE, Freetly HC. The relationship between heart rate and energy expenditure in Alpine, Angora, Boer and Spanish goat wethers consuming different quality diets at level of intake near maintenance or fasting. Small Rumin Res. 2007; 70: 183–193.

[7] Lifson N, Gordon GB, McClintock R. Measurement of total carbon dioxide production by means of D2O18. J Appl Physiol. 1955; 7: 704–710. 14381351 10.1152/jappl.1955.7.6.704

[8] Junghans P, Derno M, Gehre M, Höfling R, Kowski P, Strauch G, et al Calorimetric validation of ^13^C bicarbonate and doubly labeled water method for determining the energy expenditure in goats. Eur J Nutr (Zeitschrift für Ernährungswissenschaft). 1997; 36: 268–272. 10.1007/BF01617796 9467214

[9] Toerien CA, Sahlu T, Wong WW. Energy expenditure of Angora bucks in peak breeding season estimated with the doubly-labeled water technique. J Anim Sci. 1999;77:3096–3105. 10568482 10.2527/1999.77113096x

[10] Shaffer SA. A review of seabird energetics using the doubly labeled water method. Comp Biochem Physiol A Mol Integr Physiol. 2011; 158: 315–322. 10.1016/j.cbpa.2010.07.012 20656049

[11] Yamamoto S. Estimation of heat production from heart rate measurement of free living farm animals. Jpn Agric Res Q. 1989; 23: 134–143.

[12] Brosh A. Heart rate measurements as an index of energy expenditure and energy balance in ruminants: A review. J Anim Sci. 2007; 85: 1213–1227. 17224466 10.2527/jas.2006-298

[13] Green J. The heart rate method for estimating metabolic rate: Review and recommendations. Comp Biochem Physiol A Mol Integr Physiol. 2011; 158: 287–304. 10.1016/j.cbpa.2010.09.011 20869457

[14] Lachica M, Aguilera JF. Methods to estimate the energy expenditure of goats: From the lab to the field. Small Rumin Res. 2008; 79: 179–182.

[15] Butler PJ, Green JA, Boyd IL, Speakman JR. Measuring metabolic rate in the field: the pros and cons of the doubly labelled water and heart rate methods. Funct Ecol. 2004; 18: 168–183.

[16] Blix AS, Stromme SB, Ursin H. Additional heart rate—an indicator of psychological activation. Aerosp Med/ 1974; 45: 1219–1222. 4429063

[17] Groscolas R, Viera V, Guerin N, Handrich Y, Côté SD. Heart rate as a predictor of energy expenditure in undisturbed fasting and incubating penguins. J Exp Biol. 2010; 213: 153–160. 10.1242/jeb.033720 20008372

[18] Kovács L, Jurkovich V, Bakony M, Szenci O, Póti P, Tőzsér J. Welfare implication of measuring heart rate and heart rate variability in dairy cattle: literature review and conclusions for future research. Animal. 2014; 8: 316–330. 10.1017/S1751731113002140 24308850

[19] Von Borell E, Langbein J, Després G, Hansen S, Leterrier C, Marchant-Forde J, et al Heart rate variability as a measure of autonomic regulation of cardiac activity for assessing stress and welfare in farm animals—A review. Physiol Behav. 2007; 92: 293–316. 17320122 10.1016/j.physbeh.2007.01.007

[20] Cavagna GA, Saibene FP, Margaria R. External work in walking. J Appl Physiol. 1963; 18: 1–9. 14019429 10.1152/jappl.1963.18.1.1

[21] Kumahara H, Schutz Y, Ayabe M, Yoshioka M, Yoshitake Y, Shindo M, et al The use of uniaxial accelerometry for the assessment of physical-activity-related energy expenditure: a validation study against whole-body indirect calorimetry. Br J Nutr. 2004; 91: 235–243. 14756909 10.1079/BJN20031033

[22] Payne NL, Gillanders BM, Seymour RS, Webber DM, Snelling EP, Semmens JM. Accelerometry reveals diel patterns in field metabolic rate of giant Australian cuttlefish *Sepia apama* during breeding. J Anim Ecol. 2011; 80: 422–430. 10.1111/j.1365-2656.2010.01758.x 20880022

[23] Halsey LG, Shepard ELC, Wilson RP. Assessing the development and application of the accelerometry technique for estimating energy expenditure. Comp Biochem Physiol A Mol Integr Physiol. 2011; 158: 305–314. 10.1016/j.cbpa.2010.09.002 20837157

[24] Wilson RP, White CR, Quintana F, Halsey LG, Liebsch N, Martin GR, et al Moving towards acceleration for estimates of activity-specific metabolic rate in free-living animals: the case of the cormorant. J Anim Ecol. 2006; 75: 1081–1090. 16922843 10.1111/j.1365-2656.2006.01127.x

[25] Halsey LG, Shepard ELC, Quintana F, Laich AG, Green JA, Wilson RP. The relationship between oxygen consumption and body acceleration in a range of species. Comp Biochem Physiol A Mol Integr Physiol. 2009; 152: 197–202. 10.1016/j.cbpa.2008.09.021 18854225

[26] Gleiss AC, Wilson RP, Shepard ELC. Making overall dynamic body acceleration work: on the theory of acceleration as a proxy for energy expenditure. Methods Ecol Evol. 2011; 2: 23–33.

[27] Shepard ELC, Wilson RP, Halsey LG, Quintana F, Laich AG, Gleiss AC, et al Derivation of body motion via appropriate smoothing of acceleration data. Aquat Biol. 2008; 4: 235–241.

[28] Halsey LG, Green JA, Wilson RP, Frappell PB. Accelerometry to estimate energy expenditure during activity: best practice with data loggers. Physiol Biochem Zool. 2009; 82: 396–404. 10.1086/589815 19018696

[29] Hindle AG, Rosen DA, Trites AW. Swimming depth and ocean currents affect transit costs in Steller sea lions Eumetopias jubatus. Aquat Biol. 2010; 10: 139–148.

[30] Shepard ELC, Wilson RP, Quintana F, Laich AG, Forman DW. Pushed for time or saving on fuel: fine-scale energy budgets shed light on currencies in a diving bird. Proc R Soc Lond B Biol Sci. 2009; 276: 3149–3155. 10.1098/rspb.2009.0683 PMC2817130 19515661

[31] Wilson RP, Quintana F, Hobson VJ. Construction of energy landscapes can clarify the movement and distribution of foraging animals. Proc Roy Soc B Biol Sci. 2012; 279: 975–980. 10.1098/rspb.2011.1544 PMC3259934 21900327

[32] Japan Meteorological Agency. Climate Statistics Information. 2014. Available: http://www.data.jma.go.jp/obd/stats/etrn/index.php. Accessed 31 August 2014.

[33] Trénel P, Jensen MB, Decker EL, Skjøth F. Technical note: Quantifying and characterizing behavior in dairy calves using the IceTag automatic recording device. J Dairy Sci. 2009; 92: 3397–3401. 10.3168/jds.2009-2040 19528617

[34] Qasem L, Cardew A, Wilson A, Griffiths I, Halsey LG, Shepard ELC, et al Tri-axial dynamic acceleration as a proxy for animal energy expenditure; should we be summing values or calculating the vector?. PLoS One. 2012; 7: e31187 10.1371/journal.pone.0031187 22363576 PMC3281952

[35] Fukuhara K, Fujita M, Yamamoto S. Relationship between heart rate and heat production of goats and the difference on linear regression constants between eating and walking. Jap J Zootech Sci. 1986; 57: 978–984. (in Japanese with English Abstr.).

[36] Matsumoto T, Abo Y, Yamamoto S. Relationships between Feed Intake, Daily Gain, Heart Rate and Heat Production of Growing Holstein Heifers. Jap J Zootech Sci. 1990; 61: 230–236. (in Japanese with English Abstr.).

[37] Yamamoto S, Matsumoto T, Abo Y. Relationship between Heart Rate and Heat Production of Growing Holstein Heifers. Jap J Zootech Sci. 1990; 61: 237–240. (in Japanese with English Abstr.).

[38] SAS Institute Inc SAS/STAT User’s Guide: Version6.12. North Carolina: SAS Institute Inc; 1997

[39] Agricultural and Food Research Council. The nutrition of goats. Nutrition abstracts and reviews (Series B) AFRC Technical Committee on Responses to Nutrients, Report, vol.10. Wallingford: CAB International; 1997. pp. 793–794.

[40] Shibata M, Mukai A, Kume S. Estimation of energy expenditure in dairy heifers walking on the level and on gradients. Bulletin of the Kyushu Agricultural Experiment Station. 1981; 21: 589–609.

[41] Aharoni Y, Henkin Z, Ezra A, Dolev A, Shabtay A, Orlov A, et al Grazing behavior and energy costs of activity: A comparison between two types of cattle. J Anim Sci. 2009; 87: 2719–2731. 10.2527/jas.2008-1505 19395522

[42] Gottschall JS, Kram R. Ground reaction forces during downhill and uphill running. J Biomech. 2005; 38: 445–452. 15652542 10.1016/j.jbiomech.2004.04.023

[43] Margaria R, Margaria R. Biomechanics and energetics of muscular exercise Oxford: Clarendon Press; 1976 pp. 146.

[44] Wall J, Douglas-Hamilton I, Vollrath F. Elephants avoid costly mountaineering. Curr Biol. 2006; 16: 527–529. 10.1016/j.cub.2006.06.049 16860724

[45] Herren R, Sparti A, Aminian K, Schutz Y. The prediction of speed and incline in outdoor running in humans using accelerometry. Med Sci Sports Exerc. 1999; 31: 1053–1059. 10416569 10.1097/00005768-199907000-00020

[46] Spivey RJ, Bishop CM. Interpretation of body-mounted accelerometry in flying animals and estimation of biomechanical power. J R Soc Interface. 2013; 10: 20130404 10.1098/rsif.2013.0404 23883951 PMC3758002

[47] Taylor CR, Schmidt-Nielsen K, Raab JL. Scaling of energetic cost of running to body size in mammals. Am J Physiol. 1970; 219: 1104–1107. 5459475 10.1152/ajplegacy.1970.219.4.1104

[48] Taylor CR, Heglund NC. Energetics and mechanics of terrestrial locomotion. Annu Rev Physiol. 1982; 44: 97–107. 7041812 10.1146/annurev.ph.44.030182.000525

[49] White CR, Seymour RS. Mammalian basal metabolic rate is proportional to body mass^2/3^ . Proc Natl Acad Sci U S A. 2003; 100: 4046–4049. 12637681 10.1073/pnas.0436428100 PMC153045

[50] Commonwealth Scientific and Industrial Research Organization. Feeding Standards for Australian Livestock: Ruminants (No. 23) East Melbourne: CSIRO Publications; 1990.

[51] Osuji PO. The physiology of eating and the energy expenditure of the ruminant at pasture. J Range Manage. 1974; 27: 437–443.

[52] Brosh A, Henkin Z, Ungar ED, Dolev A, Orlov A, Yehuda Y, et al Energy cost of cows’ grazing activity: Use of the heart rate method and the Global Positioning System for direct field estimation. J Anim Sci. 2006;84: 1951–1967. 16775080 10.2527/jas.2005-315

[53] Shinde AK, Bhatta R, Sankhyan SK, Verma DL. Effect of season on thermoregulatory responses and energy expenditure of goats on semi-arid range in India. J Agric Sci. 2002; 139: 87–93.

[54] Animut G, Goetsch AL, Aiken GE, Puchala R, Detweiler G, Krehbiel CR, et al Grazing behavior and energy expenditure by sheep and goats co-grazing grass/forb pastures at three stocking rates. Small Rumin Res. 2005; 59: 191–201.

[55] Halsey LG, Shepard ELC, Hulston CJ, Venables MC, White CR, Jeukendrup AE, et al Acceleration versus heart rate for estimating energy expenditure and speed during locomotion in animals: Tests with an easy model species, *Homo sapiens* . Zoology. 2008; 111: 231–241. 10.1016/j.zool.2007.07.011 18375107

[56] McLean JA. On the calculation of heat production from open-circuit calorimetric measurements. Br J Nutr. 1972; 27: 597–600. 5031186 10.1079/bjn19720130

